# Characterization of missing data patterns and mechanisms in longitudinal composite outcome trial in rheumatoid arthritis

**DOI:** 10.1177/1759720X221114103

**Published:** 2022-09-17

**Authors:** Fowzia Ibrahim, Brian D.M. Tom, David L. Scott, Andrew Toby Prevost

**Affiliations:** Centre for Rheumatic Diseases, Department of Inflammation Biology, School of Immunology & Microbial Sciences, Faculty of Life Sciences & Medicine, King’s College London, Cutcombe Road, London SE5 9RJ, UK; MRC Biostatistics Unit, University of Cambridge, Cambridge, UK; Centre for Rheumatic Diseases, Department of Inflammation Biology, School of Immunology & Microbial Sciences, Faculty of Life Sciences & Medicine, King’s College London, London, UK; Nightingale-Saunders Clinical Trials & Epidemiology Unit, Cicely Saunders Institute of Palliative Care, Policy & Rehabilitation, Florence Nightingale Faculty of Nursing, Midwifery & Palliative Care, King’s College London, London, UK

**Keywords:** disease activity, imputation, missing completely at random, missing data, rheumatoid arthritis

## Abstract

**Background:**

Composite measures, like the Disease Activity Score for 28 joints (DAS28), are key primary outcomes in rheumatoid arthritis (RA) trials. DAS28 combines four different components in a continuous measure. When one or more of these components are missing the overall composite score is also missing at intermediate or trial endpoint assessments.

**Objectives:**

This study examined missing data patterns and mechanisms in a longitudinal RA trial to evaluate how best to handle missingness when analysing composite outcomes.

**Design:**

The Tumour-Necrosis-Factor Inhibitors against Combination Intensive Therapy (TACIT) trial was an open label, pragmatic randomized multicentre two arm non-inferiority study. Patients were followed up for 12 months, with monthly measurement of the composite outcome and its components. Active RA patients were randomized to conventional disease modifying drugs (cDMARDs) or Tumour Necrosis Factor-α inhibitors (TNFis).

**Methods:**

The TACIT trial was used to explore the extent of missing data in the composite outcome, DAS28. Patterns of missing data in components and the composite outcome were examined graphically. Longitudinal multivariable logistic regression analysis assessed missing data mechanisms during follow-up.

**Results:**

Two hundred and five patients were randomized: at 12 months 59/205 (29%) had unobserved composite outcome and 146/205 (71%) had an observed DAS28 outcome; however, 34/146 had one or more intermediate assessments missing. We observed mixed missing data patterns, especially for the missing composite outcome due to one component missing rather than patient not attending thier visit. Age and gender predicted missingness components, providing strong evidence the missing observations were unlikely to be Missing Completely at Random (MCAR).

**Conclusion:**

Researchers should undertake detailed evaluations of missing data patterns and mechanisms at the final and intermediate time points, whether or not the outcome variable is a composite outcome. In addition, the impact on treatment estimates in patients who only provide data at milestone assessments need to be assessed.

**Trial Registration ISRCTN Number:**

37438295

## Introduction

Rheumatoid arthritis (RA) is long-term autoimmune condition. Its treatment focuses on controlling the joint inflammation and preventing disease progression.^1^ Composite outcomes are widely used as primary outcome measures in RA trials. They are also used in routine practice to evaluate treatment responses. These composite indices combine several components collected at the same time to calculate a single overall score. In trials, a missing composite outcome occurs when one or more components are missing. Composite scores can be missing not only for the final assessment but also at intermediate assessments.^2^

Randomized controlled trials (RCTs) are the gold standard for evaluating the efficacy of new interventions compared with standard care or placebo treatment. In general, the primary analyses of pragmatic RCTs follow the intention-to-treat (ITT) principle; all randomized participants should be included in the analysis regardless of any departure from their original randomized group.^3^ Ideally ITT analyses require baseline and post-baseline measurements on all randomized participants to be observed at all time points. However, having some missing composite scores is inevitable, particularly in pragmatic longitudinal RCTs.^4,5^

The presence of missing data in trials leads to a loss of statistical power to detect effects through a reduction in the size of the analysed sample. Such missingness can occur differentially in each treatment arms. They may also be related to the outcome value itself. Although collected data by research teams should be consistent across multicentre trials, inevitably centres vary in the extent of missing composite outcome data with the reasons for missing often not captured in their data collection. For example, a patient may feel unwell at the time of the research assessment time point and may not wish to complete all the questionnaires but is willing to complete some of the components. When composite assessments involve components from different data sources, one or two components may not be recorded and, consequently, it may not be possible to derive an overall composite score. It is therefore important to understand and investigate the patterns and mechanisms of missingness.^6^ Identifying the pattern of missingness enables researchers to determine how systematic the process of missing observations vary between variables, which is relevant because some imputation techniques are more effective with specific types of missing data patterns. Missing data can be classified in three ways. First, missing completely at random (MCAR), when the probability of the observation being missing does not depend on observed or unobserved factors. Second, missing at random (MAR), when the probability of the observation being missing depends on some observed variables. Finally, there is missing not at random (MNAR), when missing data are related to the unobserved missing data itself, which by definition cannot be known.^7,8^ MCAR reduces the study population which can be analysed and consequently reduces the statistical power. MAR can result in biased analyses; such bias can be reduced by imputation methods or by adjusting for factors associated with the missing data. MNAR cannot be easily corrected for. In this article, we focus on the continuous composite score, Disease Activity Score of 28 joints (DAS28).^9^

Our overall aim in this study was to examine the missing data patterns and mechanisms in a longitudinal trial which used a composite assessment to evaluate treatment responses. We had two specific objectives. First, to evaluate whether differential missingness exist between trial arms in both the composite outcome and its individual components. Second, to determine how missing data over time affects the components of the composite assessment.

## Methods

### Patients

Patients were recruited from 24 rheumatology clinics in England. We included men and women aged over 18 years old with disease durations over 12 months who met the 1987 criteria for classification of RA and National Institute for Health and Care Excellence (NICE) criteria for starting biologics in England.^10^ The NICE criteria comprise, disease activity score for 28 joints > 5.1 twice over 1 month apart after treatment with methotrexate; and one other disease modifying drug. We excluded patients who were unable or unwilling to give informed consent, had not had successful results with or had contraindications to all combinations of disease modifying drugs, had contraindications to tumour necrosis factor inhibitors, had serious inter-current illness, or were taking high dose corticosteroids (>10 mg prednisolone). Ethical approval was approved by University College London Hospital research ethics committee (MREC Ref 07/Q0505/57), and all patients gave written informed consent.

### Trial design

The Tumour-Necrosis-Factor Inhibitors against Combination Intensive Therapy (TACIT) trial assessed whether intensive combinations of synthetic disease modifying drugs (cDMARDs) can achieve similar clinical benefits compared with tumour necrosis factor-α inhibitors (TNFis) in patients with active RA. The trial was an open label, pragmatic randomized multicentre two arm non-inferiority study. Patients were followed up 12 months, with monthly measurement of the composite outcome and its components. The main trial findings are published.^11,12^

### Outcome measure

The primary outcome of the main efficacy trial, which had a non-inferiority design, was reduction in the Health Assessment Questionnaires (HAQ) at 12 months. Reduction in the Disease Activity Score (28 joint counts) at 12 months was a secondary outcome measure. However, DAS28 scores and its components were measured monthly; these were used to adjust treatment intensities following the different treatment strategies in the two arms of the trial. The DAS28 score is a weighted continuous scale, which ranges between 0 and 10, the higher the score the more the disease is active. The formulae used to calculate DAS28-ESR is presented in Supplementary Material.^13,14^ This study focuses on the impact of imputation methods on the assessments of DAS28 scores.

### Statistical analyses

Proportion of patients who had an observed component were compared with those patients who had missing components of the composite at each month. Comparison between observed *versus* missing components allows us to assess whether there are differences in the explanatory variables to evaluate the missing completely at random (MCAR) assumption. The patterns of missing data in components of the composite and composite outcome were assessed graphically. We also examined differences in the pattern of missingness between treatment arms and across individual components. To assess missing data mechanisms, a missing indicator variable was created which was equal to zero if the patient had an observed component or composite outcome measure. Whereas, if the patient had not been observed (i.e. missing) at each time point, then a value of one was assigned at that particular visit. Baseline characteristics were compared using Little’s test for missing completely at random, *Li*^15^ to further test the validity of MCAR assumption.

To evaluate the validity of making the Missing at Random (MAR) assumption, multivariable Generalized Linear (Mixed-Effects) Models (GLMM) using logit link function with random intercept and slope were used. In the GLMM model treatment arm, time, treatment and time interaction, as well as other baseline variables (age, gender, ethnicity, The National Health Service (NHS) regions and disease duration) were included as covariates. All analyses was carried out using StataCorp^16^ and R Core Team.^17^

## Results

### Rate of missingness during the trial

Two hundred and five patients were randomized in the trial. Of these, 59/205 (29%) were classified as withdrawn or lost to follow-up the 12-month at primary time point. The remaining 146/205 (71%) patients were classed as ‘completers’ as they had an observed DAS28 outcome at 12 months and did not withdraw from the study. Some of these patients who completed the trial 34/146 (23%) had one or more intermediate assessments missing. This was mainly due to a mixture of non-attending the planned visit (*n* = 27) and attending but one component (ESR) missing (*n* = 7). Only 112/205 (55%) of patients had full components observed throughout the trial across all protocol visits.

When the percentage of each missing components and the DAS28 were examined over the follow up period, it was clear that the percentage of missing outcome data increased on a monthly basis throughout the trial, except at months six and twelve (Supplementary Table 1). There were large reductions in missing observations at the 6 and 12 months, which were the two important research time points. For example, at month five, the number of patients having a missing DAS28 was 23 while at month six this was reduced to 15, a reduction of 35%. Similarly, a reduction of 67% was observed in the number of missing DAS28 between 11 and 12 months (59 vs. 19). Comparable findings were shown for the individual components of the composite (Supplementary Table 1). Of the 59 patients who were classified as withdrawn or lost to follow-up at the primary time point, 19/59 were lost to follow up. The remaining 40/59 patients were ‘withdrawal but followed up’, which is defined as patients agreeing to come to the final assessment only, while allowing them to miss intermediate assessments. Hence, at primary time point (12 months), 186 patients had observed DAS28 scores (146 + 40).

We examined whether there were differences in the group of patients who had no data at 11 months (*n* = 40) compared with patients who had data at 11 months (*n* = 146). We observed higher median age difference between the groups (Mann–Whitney *p*-value = 0.029). The median age for patients without data at 11 months was 62 years (IQR: 56–68 years) compared with patients with data at 11 months, 58 years (IQR: 47–66 years) (Supplementary Table 2).

**Figure 1.**
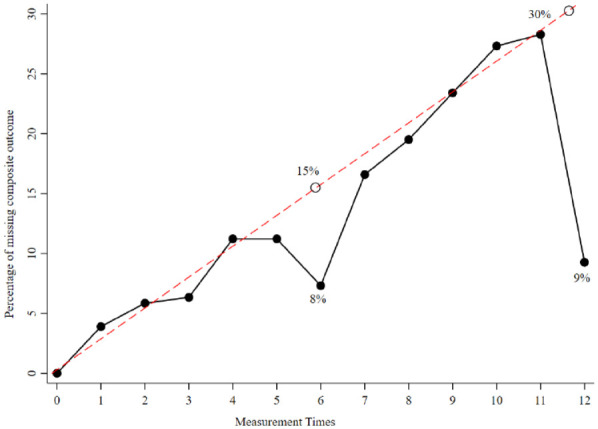
Percentage of patients with missing composite outcome at each month of follow-up. The red dash line is a simple extrapolated straight line of best fit and the black open circles represent the predicted percentage points of missing composite outcome at 6 and twelve months; solid circles are the observed percentage of missing.

Figure 1 shows a linear extrapolation of the missing percentage of the composite outcome at 6 and 12 months. The linear fit shows that the observed number of missing composite outcome at 6 and 12 months is less than expected. At 6 months, the observed missing percentage of DAS28 scores was 8% compared with the estimated percentage of 15%. Similarly, at 12 months, the observed percentage of missing DAS28 scores was 9% compared with the estimated percentage of 30%.

## Differential dropout by treatment arms

**Figure 2.**
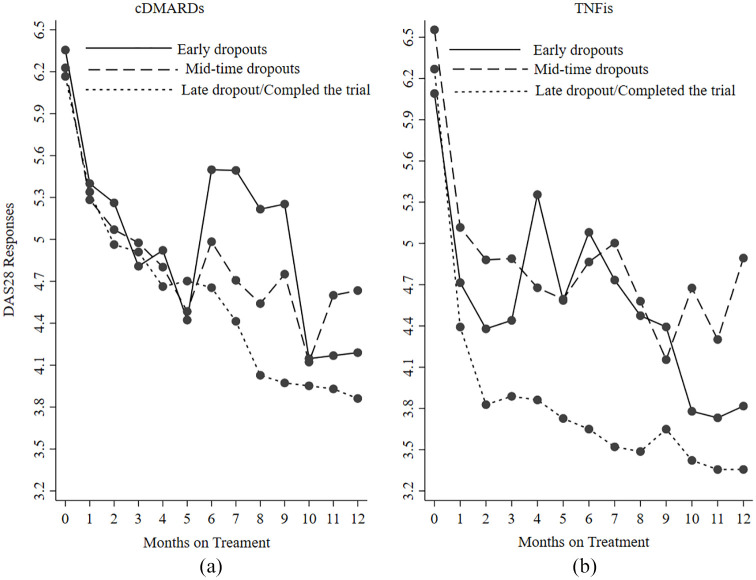
Mean profile of DAS28 responses for treatment arms stratified by time of the last assessments. cDMARDs, combination disease modifying anti rheumatic drugs; DAS28, disease activity score (28 joints); TNFis, tumour necrosis factor inhibitors; early drop out (left patients dropped out between 1 and 5 months: cDMARDs *n* = 19; TNFis *n* = 22); middle dropout (patient dropout between 6 and 10 months: cDMARDs *n* = 22; TNFis *n* = 26); late dropout/completed the trial (patients dropout/completed trial between 11 and 12 months: cDMARDs *n* = 63; TNFis *n* = 53). The observations shown after dropout are only for those patients that discontinued study intervention but agreed to be followed up at research milestone assessments.

There was a small increase in the percentage of missing components of the composite for cDMARDs against TNFis, although this was not statistically significant (Supplementary Figure 1). Furthermore, patients’ last-known assessment before dropout or completing the trial was stratified into three groups, early dropout (patient left the trial between month one and five), mid-period dropout (6–10 months) and late dropouts or completed the trial (11 and 12 months). Drop outs include both patients who discontinued the intervention but agreed to be followed up and patients who were either lost to follow or withdrew from the study. Figure 2 displays the mean DAS28 response profiles by treatment arms stratified by the length of time patients stayed in the trial. There is generally a rapid decline in DAS28 for patients who received TNFis compared with cDMARDs for early dropout patients relative to the other two groups. There were no significant differences between dropout time and the treatment arms (chi-square *p* = 0.504), though patients with early dropout in cDMARDs arm have higher mean responses than early dropouts in TNFis.

### Missing data patterns

**Figure 3.**
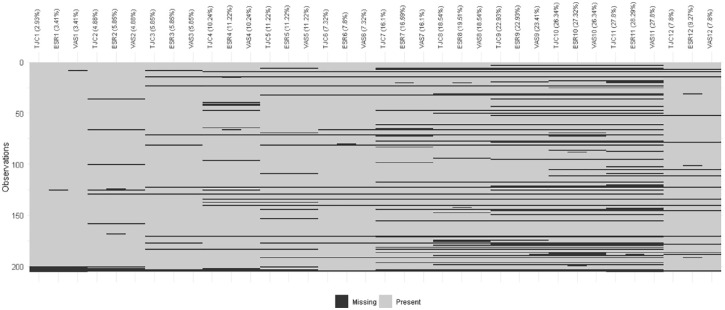
Combination of missing values in the components of the composite outcome at the follow-up time. The aggregation plot displays the different combinations of missing values (black) and non-missing values (light grey). As swollen joint counts were always present when tender joint counts were present and vice versa, these are not shown separately. The plot shows data present from 1 to 12 months for each patient; all variables were present at baseline in each patient. The percent of each variable missing at each month is shown in parenthesis. ESR, erythrocyte sedimentation rate; TJC, tender joint counts; VAS, visual analogue scale (for patient global assessments).

Figure 3 shows the combination of missing values (black) and observed values (grey) over time (horizontal axis); results for each patient are shown on the vertical axis. Over time, the proportion of missing data increased. It was minimal for the first month and maximal at month 11. The figure also shows the range of missing data patterns which span dropouts, intermittent missing data patterns (patients not attending one scheduled visit but returning for a subsequent visit) and missing components (patients attending a planned visit when not all the components were collected).

The patterns of missing observations in DAS28 are shown in Table 1. There are 74 patients who have intermittent missing patterns, of which 11/74 were due to one component missing rather than the patient not attending the planned visit. Patients who displayed intermittent missing pattern missed on average three assessments, ranging between one and ten assessments.

**Table 1.** Patterns of missingness at follow-up time by treatment arms.

Patterns	cDMARDs *n = 104*	TNFis *n = 101*	Total *N = 205*
Complete observations^a^	62 (59%)	50 (50%)	112 (55%)
Intermittent missingness due to			
Missed visit	26 (25%)	37 (36%)	63 (31%)
One component missing (ESR)	4 (4%)	7 (7%)	11 (5%)
Monotone missingness due to			
All components missing	9 (9%)	6 (6%)	15 (7%)
One component missing (ESR)	3 (3%)	1(1%)	4 (2%)

cDMARDs, combination disease modifying anti rheumatic drugs; ESR, erythrocyte sedimentation rate; TNFis, tumour necrosis factor inhibitors.

a Patients attended all the visits and have four components are observed throughout the 12 months period.

### Predictors of missing data mechanisms

There was a notable difference in these outcomes between those patients with missing observations and those without missing observations (Supplementary Table 3). Patients without missing observations in the components and composite outcome were younger, with lower mean disease duration. Furthermore, males were more likely to drop out from the trial than females. There were no significant differences between patients with and without observations in treatment arms, ethnicity or NHS-region.

**Figure 4.**
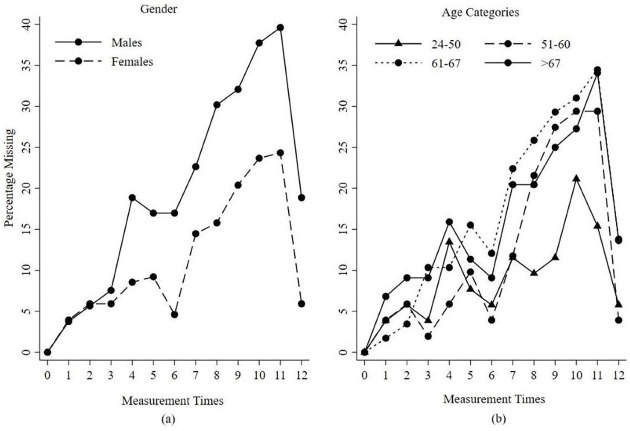
Percentage of patients with missing composite outcome by age group and gender at follow-up time points, (a) Gender; (b) Age Categories.

Figure 4 shows the percentage of missing DAS28 during the trial stratified by gender (a) and age groups (b). Patients’ age was categorized into quantiles to have equal numbers in each category. It confirms that the older the patients are the more likely they are to dropout from the trial. Similarly, males were more likely to drop out from the trial than females.

Table 2 presents the odds ratios from the longitudinal logistic regressions of the indicator missing components and composite outcome on baseline covariates. The results show that the data are unlikely to be missing under MCAR assumption as age and gender were associated with missingness in the multivariate model. Males were more likely to have missing DAS28 than females [OR: 3.29 (95% CI: 1.38, 7.86), *p* = 0.007]. Likewise, older patients were more likely to have missing DAS28 [OR: 1.04 (95% CI: 1.01, 1.08), *p* = 0.019]. The components of composite outcome show similar findings. There was no evidence of multiplicative interactions between treatment arm and time in the multivariable model (all *p*-values were > 0.05). In addition to the longitudinal logistic regression models, the Little’s MCAR test for components was performed. The finding showed that the missingness in the tender joint counts, ESR and VAS were statistically significant at the 5%, level all *p* < 0.05. For the DAS28 composite outcome, the chi-square *p*-value was 0.0149, which provides strong evidence that the missing DAS28 observations are not MCAR.

**Table 2.** Longitudinal logistic models for the factors influencing missingness in each of the component and composite outcome in TACIT trial.

	Missing outcome data on tender and swollen joint counts	Missing data on ESR	Missing data on VAS	Missing data on DAS28
	*OR (95% CI)*	*OR (95% CI)*	*OR (95% CI)*	*OR (95% CI)*
Treatment arms				
cDMARDs	Reference	Reference	Reference	Reference
TNF Inhibitors	1.15 (0.49, 2.70)	1.19 (0.54, 2.62)	1.15 (0.49, 2.69)	1.17(0.53, 2.59)
Gender				
Female	Reference	Reference	Reference	Reference
Male	3.21 (1.26, 8.17)	3.03 (1.27, 7.23)	3.19 (1.25, 8.11)	3.29 (1.38, 7.86)
Ethnicity				
White	Reference	Reference	Reference	Reference
Other	1.49 (0.37, 6.11)	1.30 (0.34, 4.87)	1.59 (0.39, 6.46)	1.34 (0.36, 5.03)
The National Health Service (NHS) Region				
London and South England	Reference	Reference	Reference	Reference
Midlands	3.76 (0.84, 16.74)	3.95 (0.98, 15.89)	3.91 (0.88, 17.39)	4.02 (0.99, 16.22)
North England	1.76 (0.69, 4.50)	1.77 (0.74, 4.20)	1.76 (0.69, 4.49)	1.63 (0.68, 3.89)
Age	1.05 (1.01, 1.09)	1.05 (1.01, 1.09)	1.05 (1.01, 1.09)	1.04 (1.01, 1.08)
Disease duration	0.97 (0.92, 1.02)	0.98 (0.93, 1.03)	0.97 (0.92, 1.02)	0.98 (0.94, 1.03)

cDMARDs, combination disease modifying anti rheumatic drugs; 95 % CI, 95% confidence interval; DAS28, disease activity score (28 joints); ESR, erythrocyte sedimentation rate; OR, odds ratio; TACIT, Tumour-Necrosis-Factor Inhibitors against Combination Intensive Therapy; TNF, Tumour necrosis factor inhibitors; VAS, visual analogue scale.

Odds ratio (OR) represent odds of having missing component or composite over the trial follow-up

## Discussion

In the TACIT trial, levels of missingness were similar in both trial arms. Some patients were more likely to have missingness, which was highest amongst older and male patients. As the research team invested considerable time and effort to ensure patients attended at the key research assessment time points, the true dropout rate is masked at the primary endpoint. The percentage of missingness in the components and composite outcome increased month by month as the trial progressed, but at the primary trial endpoint missingness was substantially reduced. For example, at month 11, there were 59 patients missing DAS28 observations. While at month 12, there were only 19 patients with missing DAS28 data. This difference shows participating centres focussed on getting patients back for their final assessment while overlooking the intermediate time points in order to ensure higher attendances at key milestones. By taking this pragmatic step, the research team reduced the number of older patients who would otherwise be lost from the trial. However, the protocol required DAS28 scores to be available at each visit so that treatment could be adjusted appropriately accordingly.^11,12^ As a consequence of missing data some patients might have not had their treatment changed at all,^12^ or they might have had treatment changes based on inappropriate DAS28 scores calculated using some data from an earlier visit.

In the TACIT trial, the DAS28 scores were used to monitor treatment responses so that medications could be adjusted in line with clinical practice at the time. The primary outcome measure was changes over 12 months in disability scores measured using the Health Assessment Questionnaire (HAQ) in a non-inferiority design. Consequently, issues about missingness of DAS28 scores would not necessarily have impacted on the trial outcome. Nevertheless, as DAS28 is widely used as the primary outcome measure in many RA trials the issues of missingness are important considerations for trial design and analysis.

In our analysis, making an MCAR assumption would have been unrealistic as the results show the probability of component being missing was dependent on age and gender. Any treatment estimates from an MCAR analysis are likely to be biased, especially as gender and age are predictive of outcome.^3^ Therefore, it is more plausible to make MAR assumption than an MCAR, although missingness could potentially have been MNAR White *et al.*^18^ and sensitivity analysis would be required to support the robustness of the primary analysis. In addition, many patients had at least one or more measurements recorded during follow-up, suggesting that a sensible imputation approach to explore the longitudinal data structure is Multiple Imputation with chained equation (MICE),^19^ while incorporating the partially available measurements. Several detailed accounts of appropriate multiple imputation methods are available for different clinical settings including online advice from Van Buuren^20^ and guidance from Mainzer *et al.*^21^

Two limitations of our analyses require further consideration. First, we only considered a dataset from a single trial. It is highly likely that in other trials in RA, using the DAS28, there may be different patterns and mechanisms of missingness. Consequently, our results must be used cautiously when extrapolating to other trials. Second, the low drop out rate in the TACIT trial may be misleading. Although only 8% (16/205) of patients had no end-point data, all components of their DAS28 scores were only measured throughout the trial in 55% of patients. Ignoring missing intermediate measures for some patients in terms of drop outs in TACIT overlooks the potential impact of missing composite outcome data, especially as the composite outcome was used to guide treatment decisions in the trial. Consequently, comparing drop outs and missingness of data in TACIT with other trials that might have higher end-point drop outs is challenging.

## Conclusion

In conclusion, we believe researchers should use appropriate methods to impute missing data in trials, and that their general approach should be included within the trial protocol and statistical analysis plan, in line with guidance from Jakobsen *et al.*^22^ We also recommend researchers should undertake a detailed evaluation of missing data patterns and mechanisms at the final and intermediate time points, whether the outcome variable is a composite outcome or not.

## Supplemental Material

sj-docx-1-tab-10.1177_1759720X221114103 – Supplemental material for Characterization of missing data patterns and mechanisms in longitudinal composite outcome trial in rheumatoid arthritis Click here for additional data file. Supplemental material, sj-docx-1-tab-10.1177_1759720X221114103 for Characterization of missing data patterns and mechanisms in longitudinal composite outcome trial in rheumatoid arthritis by Fowzia Ibrahim, Brian D.M. Tom, David L. Scott and Andrew Toby Prevost in Therapeutic Advances in Musculoskeletal Disease

## Declarations

**Ethics approval and consent to participate:** This study was approved by University College London Hospital research ethics committee (reference number: 07/Q0505/57). Informed written consent was obtained.

**Consent for publication:** Not applicable.

**Availability of data and materials:** The dataset analysed is not publicly available because data sharing was not part of the original consent and requires institutional approval, but data requests should be submitted to the corresponding author and summary data may be granted following review.
