# Supplementary material for: Characterization of missing data patterns and mechanisms in longitudinal composite outcome trial in rheumatoid arthritis
Source: Ther Adv Musculoskelet Dis. 2022 Sep 17;14:1759720X221114103. doi: 10.1177/1759720X221114103 (PMC9486257; doi:10.1177/1759720X221114103)
Supplement: sj-docx-1-tab-10.1177_1759720X221114103 – Supplemental material for Characterization of missing data patterns and mechanisms in longitudinal composite outcome trial in rheumatoid arthritis [file sj-docx-1-tab-10.1177_1759720X221114103.docx]

**Supplementary Material**

Calculating Disease Activity Score (28 joints)

Variables used to calculate the DAS28 are:

- Counts of the numbers of joints (out of 28) that are tender and the number of swollen (in hands, arms, and knees)
- Erythrocyte Sedimentation Rate (ESR) which is measured in mm/hour, is a blood test that measures the amount of inflammation in the body
- Visual Analogue Scale (VAS), measure of disease activity assessed by patients using a 100 mm scale, which ranges between 0-100mm; 0=not active at all and 100=extremely active

The DAS28 score is a weighted continuous scale, which ranges between 0 and 10, the higher the score the more the disease is active.

The formulae below are known as the 4-variable DAS for ESR, which combines the 4-components by weighting and summation.


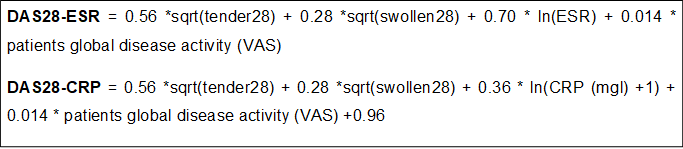


One of the advantages of DAS28 is a simple measure to use in clinical practice. However, the main disadvantage is the validity of the measures that are chosen to be included and the suitability of their relative weighting^1,2^. The overall score needs a careful interpretation because a high value of DAS28 can reflect high pain levels or active arthritis or both^2^.

Supplementary Table 1: Number and Percent of missing observations in the composite outcome and its components at each month of follow-up (n=205)

| Measurement Time in Months | TJC | SJC | ESR | VAS | DAS28 |
| --- | --- | --- | --- | --- | --- |
|  | n (%) | n (%) | n (%) | n (%) | n (%) |
| Baseline | 0 | 0 | 0 | 0 | 0 |
| 1 | 6 (2.9%) | 6 (2.9%) | 7 (3.4%) | 7 (3.4%) | 8 (3.9%) |
| 2 | 10 (4.9%) | 10 (4.9%) | 12 (5.9%) | 10 (4.9%) | 12 (5.9%) |
| 3 | 12 (5.9%) | 12 (5.9%) | 13 (6.3%) | 12 (5.9%) | 13 (6.3%) |
| 4 | 21 (10.2%) | 21 (10.2%) | 23 (11.2%) | 21 (10.2%) | 23 (11.2%) |
| 5 | 23 (11.2%) | 23 (11.2%) | 23 (11.2%) | 23 (11.2%) | 23 (11.2%) |
| 6 | 15 (7.3%) | 15 (7.3%) | 16 (7.8%) | 15 (7.3%) | 16 (7.8%) |
| 7 | 33 (16.1%) | 33 (16.1%) | 34 (16.6%) | 33 (16.1%) | 34 (16.6%) |
| 8 | 38 (18.5%) | 38 (18.5%) | 40 (19.5%) | 38 (18.5%) | 40 (19.5%) |
| 9 | 47 (22.9%) | 47 (22.9%) | 47 (22.9%) | 48 (23.4%) | 48 (23.4%) |
| 10 | 54 (26.3%) | 54 (26.3%) | 56 (27.3%) | 54 (26.3%) | 56 (27.3%) |
| 11 | 57 (27.8%) | 57 (27.8%) | 59 (28.3%) | 57 (27.8%) | 59 (28.3%) |
| 12 | 16 (7.8%) | 16 (7.8%) | 19 (9.3%) | 16 (7.8%) | 19 (9.3%) |

TJC=tender joint counts; SJC=swollen joint counts; ESR=erythrocyte sedimentation rate; VAS=visual analogue scale; DAS28=disease activity score (28 joints)

Supplementary Table 2: Baseline demographic and clinical characteristics of patients with and without data at eleven months visit

|  | **No data at 11 months** | **Data at 11 months** | **Data available at the primary time point** |
| --- | --- | --- | --- |
|  | (N = 40) | (N = 146) | (N = 186) |
| **Demographic variables** | Median (IQR) | Median (IQR) | Median (IQR) |
| Age in years | 61.5 (55.5, 67.5) | 57.5 (47.0, 66.0) | 59.5 (49.0 ,66.0) |
| Disease duration in years | 2.3 (1.5, 11.4) | 5.1 (2.1, 11.3) | 4.8 (2.0 ,11.3) |
| Gender, n (%) |  |  |  |
| Male | 11 (27.5%) | 32 (21.9%) | 53 (25.9%) |
| Female | 29 (72.5%) | 114 (78.1%) | 152 (74.1%) |
| Ethnicity, n (%) |  |  |  |
| White | 36 (90.0%) | 126 (86.3%) | 181 (88.3%) |
| Other | 4 (10.0%) | 20 (13.7%) | 24 (11.7%) |
| Region, n (%) |  |  |  |
| London & South | 24 (60.0%) | 98 (67.1%) | 128 (62.4%) |
| Midlands | 4 (10.0%) | 6 (4.1%) | 16 (7.8%) |
| North | 12 (30.0%) | 42 (28.8%) | 61 (29.8%) |
| Randomised group |  |  |  |
| cDMARDs | 21 (52.5%) | 71 (48.6%) | 92 (49.5%) |
| TNFis | 19 (47.5%) | 75 (51.4%) | 94 (50.5%) |
| **Clinical variables at baseline** |  |  |  |
| Joint counts | 14.5 (11.5, 23.5) | 17.0 (12.0, 23.0) | 16.0 (12.0 ,23.0) |
| ESR | 22.5 (9.5, 61.0) | 27.0 (14.0, 40.0) | 26.5 (13.0 ,41.0) |
| PGA | 72.5 (53.5, 85.5) | 70.0 (53.0, 84.0) | 70.5 (53.0 ,84.0) |
| DAS28-ESR | 6.3 (5.7, 6.8) | 6.2 (5.6, 6.8) | 6.3 (5.7 ,6.8) |

ESR= Erythrocyte Sedimentation Rate (mm/hr; PGA= Patient Global Assessment (mm); DAS28= Disease Activity Score for 28 Joints based on ESR; cDMARDs = combination disease modifying anti rheumatic drugs; TNFis= Tumor necrosis factor inhibitors;

Supplementary Table 3: Baseline characteristics influencing missingness in the components and continuous composite outcome in TACIT trial

|  | **Missing** | | |  | | | **Tender Joint Counts** | | | | | | | | | | | | | | | | | | | | | | | | | | | | | | | | | | | | | | | | | | |
| --- | --- | --- | --- | --- | --- | --- | --- | --- | --- | --- | --- | --- | --- | --- | --- | --- | --- | --- | --- | --- | --- | --- | --- | --- | --- | --- | --- | --- | --- | --- | --- | --- | --- | --- | --- | --- | --- | --- | --- | --- | --- | --- | --- | --- | --- | --- | --- | --- | --- |
|  |  |  |  | No  Visits | | | Age (years) | | | | Disease duration  (years) | | | Treatment | | | | | | | | Gender | | | | | | | | Ethnicity | | | | | | | NHS-Region | | | | | | | | | | | | |
|  |  |  |  |  | | | Mean (SD) | | | | Mean (SD) | | | cDMARds  n(%) | | | | TNF  n(%) | | | | Male  n (%) | | | | Female  n (%) | | | | White  n (%) | | | | Other  n(%) | | | London & South  n(%) | | | | | Midlands  n(%) | | | North  n(%) | | | | |
|  |  |  |  | 1 | | | 58.0 (13.5) | | | | 7.2 (9.8) | | | 2 (1.92) | | | | 4 (3.96) | | | | 2 (3.77) | | | | 4 (2.63) | | | | 6 (3.31) | | | | 0 | | | 4 (3.13) | | | | | 1 (6.25) | | | 1 (1.64) | | | | |
|  |  |  |  | 2 | | | 58.3 (12.4) | | | | 8.7 (9.8) | | | 5 (4.81) | | | | 5 (4.95) | | | | 3 (5.66) | | | | 7 (4.61) | | | | 9 (4.97) | | | | 1 (4.17) | | | 8 (6.25) | | | | | 1 (6.25) | | | 1 (1.64) | | | | |
|  |  |  |  | 3 | | | 62.6 (9.8) | | | | 6.0 (5.8) | | | 8 (7.69) | | | | 4 (3.96) | | | | 3 (5.66) | | | | 9 (5.92) | | | | 11(6.08) | | | | 1 (4.17) | | | 3 (2.34) | | | | | 1 (6.25) | | | 8 (13.11) | | | | |
|  |  |  |  | 4 | | | 60.1 (13.1) | | | | 10.3 (8.8) | | | 12 (11.54) | | | | 9 (8.91) | | | | 8 (15.09) | | | | 13 (8.55) | | | | 19 (10.50) | | | | 2 (8.33) | | | 10 (7.81) | | | | | 2 (12.50) | | | 9 (14.75) | | | | |
|  |  |  |  | 5 | | | 59.8 (10.9) | | | | 7.6 (6.8) | | | 12 (11.54) | | | | 11 (10.89) | | | | 9 (16.98) | | | | 14 (9.21) | | | | 20 (11.05) | | | | 3 (12.50) | | | 8 (6.25) | | | | | 4 (25.00) | | | 11 (18.03) | | | | |
|  |  |  |  | 6 | | | 60.3 (12.0) | | | | 5.8 (6.3) | | | 10 (9.62) | | | | 5 (4.95) | | | | 8 (15.09) | | | | 7 (4.61) | | | | 14 (7.73) | | | | 1 (4.17) | | | 1 (0.78) | | | | | 3 (18.75) | | | 11 (18.03) | | | | |
|  |  |  |  | 7 | | | 60.8 (11.0) | | | | 6.2 (6.1) | | | 18 (17.31) | | | | 15 (14.85) | | | | 12 (22.64) | | | | 21 (13.82) | | | | 29 (16.02) | | | | 4 (16.67) | | | 16 (12.50) | | | | | 4 (25.00) | | | 13 (21.31) | | | | |
|  |  |  |  | 8 | | | 60.7 (10.2) | | | | 5.8 (5.8) | | | 21 (20.19) | | | | 17 (16.83) | | | | 16 (30.19) | | | | 22 (14.47) | | | | 34 (18.78) | | | | 4 (16.67) | | | 18 (14.06) | | | | | 5 (31.25) | | | 15 (24.59) | | | | |
|  |  |  |  | 9 | | | 60.9 (10.0) | | | | 7.5 (8.5) | | | 26 (25.00) | | | | 21 (20.79) | | | | 17 (32.08) | | | | 30 (19.74) | | | | 43 (23.76) | | | | 4 (16.67) | | | 22 (17.19) | | | | | 7 (43.75) | | | 18 (29.51) | | | | |
|  |  |  |  | 10 | | | 60.0 (10.8) | | | | 6.8 (7.7) | | | 29 (27.88) | | | | 25 (24.75) | | | | 19 (35.85) | | | | 35 (23.03) | | | | 48 (26.52) | | | | 6 (25.00) | | | 29 (22.66) | | | | | 8 (50.00) | | | 17 (27.87) | | | | |
|  |  |  |  | 11 | | | 61.3 (10.0) | | | | 7.5 (8.8) | | | 32 (30.77) | | | | 25 (24.75) | | | | 21 (39.62) | | | | 36 (23.68) | | | | 53 (29.28) | | | | 4 (16.67) | | | 30 (23.44) | | | | | 8 (50.00) | | | 19 (31.15) | | | | |
|  |  |  |  | 12 | | | 62.3 (10.8) | | | | 8.2 (8.8) | | | 9 (8.65) | | | | 7 (6.93) | | | | 8 (15.09) | | | | 8 (5.26) | | | | 16 (8.84) | | | | 0 | | | 5 (3.91) | | | | | 4 (25.00) | | | 7 (11.48) | | | | |
|  | **Non-Missing** | | | 1 | | | 57.3 (12.0) | | | | 8.2 (8.8) | | | 102 (98.08) | | | | 97 (96.04) | | | | 51 (96.23) | | | | 148 (97.37) | | | | 175 (96.69) | | | | 24 (100) | | | 124 (96.88) | | | | | 15 (93.75) | | | 60 (98.36) | | | | |
|  |  |  |  | 2 | | | 57.3 (12.0) | | | | 8.2 (8.8) | | | 99 (95.19) | | | | 96 (95.05) | | | | 50 (94.34) | | | | 145 (95.39) | | | | 172 (95.03) | | | | 23 (95.83) | | | 120 (93.75) | | | | | 15 (93.75) | | | 60 (98.36) | | | | |
|  |  |  |  | 3 | | | 57.0 (12.0) | | | | 8.3 (9.0) | | | 96 (92.31) | | | | 97 (96.04) | | | | 50 (94.34) | | | | 143 (94.08) | | | | 170 (93.92) | | | | 23 (95.83) | | | 125 (97.66) | | | | | 15 (93.75) | | | 53 (86.89) | | | | |
|  |  |  |  | 4 | | | 57.0 (11.8) | | | | 8.0 (8.8) | | | 92 (88.46) | | | | 92 (91.09) | | | | 45 (84.91) | | | | 139 (91.45) | | | | 162 (89.50) | | | | 22 (91.67) | | | 118 (92.19) | | | | | 14 (87.50) | | | 52 (85.25) | | | | |
|  |  |  |  | 5 | | | 57.0 (12.1) | | | | 8.3 (9.1) | | | 92 (88.46) | | | | 90 (89.11) | | | | 44 (83.02) | | | | 138 (90.79) | | | | 161 (88.95) | | | | 21 (87.50) | | | 120 (93.75) | | | | | 12 (75.00) | | | 50 (81.97) | | | | |
|  |  |  |  | 6 | | | 57.1 (12.0) | | | | 8.4 (9.0) | | | 94 (90.38) | | | | 96 (95.05) | | | | 45 (84.91) | | | | 145 (95.39) | | | | 167 (92.27) | | | | 23 (95.83) | | | 127 (99.22) | | | | | 13 (81.25) | | | 50 (81.97) | | | | |
|  |  |  |  | 7 | | | 56.7 (12.1) | | | | 8.6 (9.2) | | | 86 (82.69) | | | | 86 (85.15) | | | | 41 (77.36) | | | | 131 (86.18) | | | | 152 (83.98) | | | | 20 (83.33) | | | 112 (87.50) | | | | | 12 (75.00) | | | 48 (78.69) | | | | |
|  |  |  |  | 8 | | | 56.6 (12.2) | | | | 8.7 (9.3) | | | 83 (79.81) | | | | 84 (83.17) | | | | 37 (69.81) | | | | 130 (85.53) | | | | 147 (81.22) | | | | 20 (83.33) | | | 110 (85.94) | | | | | 11 (68.75) | | | 46 (75.41) | | | | |
|  |  |  |  | 9 | | | 56.3 (12.3) | | | | 8.4 (9.0) | | | 78 (75.00) | | | | 80 (79.21) | | | | 36 (67.92) | | | | 122 (80.26) | | | | 138 (76.24) | | | | 20 (83.33) | | | 106 (82.81) | | | | | 9 (56.25) | | | 43 (70.49) | | | | |
|  |  |  |  | 10 | | | 56.4 (12.3) | | | | 8.7 (9.2) | | | 75 (72.12) | | | | 76 (75.25) | | | | 34 (64.15) | | | | 117 (76.97) | | | | 133 (73.48) | | | | 18 (75.00) | | | 99 (77.34) | | | | | 8 (50.00) | | | 44 (72.13) | | | | |
|  |  |  |  | 11 | | | 55.8 (12.3) | | | | 8.5 (8.9) | | | 72 (69.23) | | | | 76 (75.25) | | | | 32 (60.38) | | | | 116 (76.32) | | | | 128 (70.72) | | | | 20 (83.33) | | | 98 (76.56) | | | | | 8 (50.00) | | | 42 (68.85) | | | | |
|  |  |  |  | 12 | | | 56.9 (12.0) | | | | 8.2 (8.9) | | | 95 (91.35) | | | | 94 (93.07) | | | | 45 (84.91) | | | | 144 (94.74) | | | | 165 (91.16) | | | | 24 (100) | | | 123 (96.09) | | | | | 12(75.00) | | | 54 (88.52) | | | | |
|  | |  | | | **Swollen Joint Counts** | | | | | | | | | | | | | | | | | | | | | | | | | | | | | | | | | | | | | | | | | | | |  |
| **Missing** | | N^o^ Visits | | | Age (years) | | | | | Disease duration  (years) | | | Treatment | | | | | | | | Gender | | | | | | | | Ethnicity | | | | | | | | | NHS-Region | | | | | | | | | | |  |
|  |  |  | | | Mean (SD) | | | | | Mean (SD) | | | cDMARds  n(%) | | | | TNF  n(%) | | | | Male  n(%) | | | | Female  n(%) | | | | White  n(%) | | | | Other  n(%) | | | | | London & South  n(%) | | Midlands  n(%) | | | North  n(%) | | | | | |  |
|  |  | 1 | | | 58.0 (13.5) | | | | | 7.2 (9.8) | | | 2 (1.92) | | | | 4 (3.96) | | | | 2 (3.77) | | | | 4 (2.63) | | | | 6 (3.31) | | | | 0 | | | | | 4 (3.13) | | 1 (6.25) | | | 1 (1.64) | | | | | |  |
|  |  | 2 | | | 58.3 (12.4) | | | | | 8.7 (9.8) | | | 5 (4.81) | | | | 5 (4.95) | | | | 3 (5.66) | | | | 7 (4.61) | | | | 9 (4.97) | | | | 1 (4.17) | | | | | 8 (6.25) | | 1 (6.25) | | | 1 (1.64) | | | | | |  |
|  |  | 3 | | | 62.6 (9.8) | | | | | 6.0 (5.8) | | | 8 (7.69) | | | | 4 (3.96) | | | | 3 (5.66) | | | | 9 (5.92) | | | | 11 (6.08) | | | | 1 (4.17) | | | | | 3(2.34) | | 1 (6.25) | | | 8 (13.11) | | | | | |  |
|  |  | 4 | | | 60.1 (13.1) | | | | | 10.3 (8.8) | | | 12 (11.54) | | | | 9 (8.91) | | | | 8 (15.09) | | | | 13 (8.55) | | | | 19 (10.50) | | | | 2 (8.33) | | | | | 10 (7.81) | | 2 (12.50) | | | 9 (14.75) | | | | | |  |
|  |  | 5 | | | 59.8 (10.9) | | | | | 7.6 (6.8) | | | 12 (11.54) | | | | 11 (10.89) | | | | 9 (16.98) | | | | 14 (9.21) | | | | 20 (11.05) | | | | 3 (12.50) | | | | | 8 (6.25) | | 4 (25.00) | | | 11 (18.03) | | | | | |  |
|  |  | 6 | | | 60.3 (12.0) | | | | | 5.8 (6.3) | | | 10 (9.62) | | | | 5 (4.95) | | | | 8 (15.09) | | | | 7 (4.61) | | | | 14 (7.73) | | | | 1 (4.17) | | | | | 1 (0.78) | | 3 (18.75) | | | 11 (18.03) | | | | | |  |
|  |  | 7 | | | 60.8 (11.0) | | | | | 6.2 (6.1) | | | 18 (17.31) | | | | 15 (14.85) | | | | 12 (22.64) | | | | 21 (13.82) | | | | 29 (16.02) | | | | 4 (16.67) | | | | | 16 (12.50) | | 4 (25.00) | | | 13 (21.31) | | | | | |  |
|  |  | 8 | | | 60.7 (10.2) | | | | | 5.8 (5.8) | | | 21 (20.19) | | | | 17 (16.83) | | | | 16 (30.19) | | | | 22 (14.47) | | | | 34 (18.78) | | | | 4 (16.67) | | | | | 18 (14.06) | | 5 (31.25) | | | 15 (24.59) | | | | | |  |
|  |  | 9 | | | 60.9 (10.0) | | | | | 7.5 (8.5) | | | 26 (25.00) | | | | 21 (20.79) | | | | 17 (32.08) | | | | 30 (19.74) | | | | 43 (23.76) | | | | 4 (16.67) | | | | | 22 (17.19) | | 7 (43.75) | | | 18 (29.51) | | | | | |  |
|  |  | 10 | | | 60.0 (10.8) | | | | | 6.8 (7.7) | | | 29 (27.88) | | | | 25 (24.75) | | | | 19 (35.85) | | | | 35 (23.03) | | | | 48 (26.52) | | | | 6 (25.00) | | | | | 29 (22.66) | | 8 (50.00) | | | 17 (27.87) | | | | | |  |
|  |  | 11 | | | 61.3 (10.0) | | | | | 7.5 (8.8) | | | 32 (30.77) | | | | 25 (24.75) | | | | 21 (39.62) | | | | 36 (23.68) | | | | 53 (29.28) | | | | 4 (16.67) | | | | | 30 (23.44) | | 8 (50.00) | | | 19 (31.15) | | | | | |  |
|  |  | 12 | | | 62.3 (10.8) | | | | | 8.2 (8.8) | | | 9 (8.65) | | | | 7 (6.93) | | | | 8 (15.09) | | | | 8 (5.26) | | | | 16 (8.84) | | | | 0 | | | | | 5 (3.91) | | 4 (25.00) | | | 7 (11.48) | | | | | |  |
| **Non-Missing** | | 1 | | | 57.3 (12.0) | | | | | 8.2 (8.8) | | | 102 (98.08) | | | | 97 (96.04) | | | | 51 (96.23) | | | | 148 (97.37) | | | | 175 (96.69) | | | | 24 (100) | | | | | 124 (96.88) | | 15(93.75) | | | 60 (98.36) | | | | | |  |
|  |  | 2 | | | 57.3 (12.0) | | | | | 8.2 (8.8) | | | 99 (95.19) | | | | 96 (95.05) | | | | 50 (94.34) | | | | 145 (95.39) | | | | 172 (95.03) | | | | 23 (95.83) | | | | | 120 (93.75) | | 15(93.75) | | | 60 (98.36) | | | | | |  |
|  |  | 3 | | | 57.0 (12.0) | | | | | 8.3 (9.0) | | | 96 (92.31) | | | | 97 (96.04) | | | | 50 (94.34) | | | | 143 (94.08) | | | | 170 (93.92) | | | | 23 (95.83) | | | | | 125 (97.66) | | 15(93.75) | | | 53 (86.89) | | | | | |  |
|  |  | 4 | | | 57.0 (11.8) | | | | | 8.0 (8.8) | | | 92 (88.46) | | | | 92 (91.09) | | | | 45 (84.91) | | | | 139 (91.45) | | | | 162 (89.50) | | | | 22 (91.67) | | | | | 118 (92.19) | | 14(87.50) | | | 52 (85.25) | | | | | |  |
|  |  | 5 | | | 57.0 (12.1) | | | | | 8.3 (9.1) | | | 92 (88.46) | | | | 90 (89.11) | | | | 44 (83.02) | | | | 138 (90.79) | | | | 161 (88.95) | | | | 21 (87.50) | | | | | 120 (93.75) | | 12(75.00) | | | 50 (81.97) | | | | | |  |
|  |  | 6 | | | 57.1 (12.0) | | | | | 8.4 (9.0) | | | 94 (90.38) | | | | 96 (95.05) | | | | 45 (84.91) | | | | 145 (95.39) | | | | 167 (92.27) | | | | 23 (95.83) | | | | | 127 (99.22) | | 13(81.25) | | | 50 (81.97) | | | | | |  |
|  |  | 7 | | | 56.7 (12.1) | | | | | 8.6 (9.2) | | | 86 (82.69) | | | | 86 (85.15) | | | | 41 (77.36) | | | | 131 (86.18) | | | | 152 (83.98) | | | | 20 (83.33) | | | | | 112 (87.50) | | 12(75.00) | | | 48 (78.69) | | | | | |  |
|  |  | 8 | | | 56.6 (12.2) | | | | | 8.7 (9.3) | | | 83 (79.81) | | | | 84 (83.17) | | | | 37 (69.81) | | | | 130 (85.53) | | | | 147 (81.22) | | | | 20 (83.33) | | | | | 110 (85.94) | | 11(68.75) | | | 46 (75.41) | | | | | |  |
|  |  | 9 | | | 56.3 (12.3) | | | | | 8.4 (9.0) | | | 78 (75.00) | | | | 80 (79.21) | | | | 36 (67.92) | | | | 122 (80.26) | | | | 138 (76.24) | | | | 20 (83.33) | | | | | 106 (82.81) | | 9(56.25) | | | 43 (70.49) | | | | | |  |
|  |  | 10 | | | 56.4 (12.3) | | | | | 8.7 (9.2) | | | 75 (72.12) | | | | 76 (75.25) | | | | 34 (64.15) | | | | 117 (76.97) | | | | 133 (73.48) | | | | 18 (75.00) | | | | | 99 (77.34) | | 8(50.00) | | | 44 (72.13) | | | | | |  |
|  |  | 11 | | | 55.8 (12.3) | | | | | 8.5 (8.9) | | | 72 (69.23) | | | | 76 (75.25) | | | | 32 (60.38) | | | | 116 (76.32) | | | | 128 (70.72) | | | | 20 (83.33) | | | | | 98 (76.56) | | 8(50.00) | | | 42 (68.85) | | | | | |  |
|  |  | 12 | | | 56.9 (12.0) | | | | | 8.2 (8.9) | | | 95 (91.35) | | | | 94 (93.07) | | | | 45 (84.91) | | | | 144 (94.74) | | | | 165 (91.16) | | | | 24 (100) | | | | | 123 (96.09) | | 12(75.00) | | | 54 (88.52) | | | | | |  |
|  | |  | | |  | | | | | | | | | | | | | | | | | | | | | | | | | | | | | | | | | | | | | | | | | |  |  |  |
|  | |  | | | **ESR** | | | | | | | | | | | | | | | | | | | | | | | | | | | | | | | | | | | | | | | | | |  |  |  |
| **Missing** | | N^o^ Visits | | | Age (years) | | | | | Disease duration  (years) | | | Treatment | | | | | | | | Gender | | | | | | | | Ethnicity | | | | | | | | | NHS- Region | | | | | | | | |  |  |  |
|  |  |  | | | Mean (SD) | | | | | Mean (SD) | | | cDMARds  n (%) | | | | TNF  n (%) | | | | Male  n(%) | | | | Female  n(%) | | | | White  n (%) | | | | Other  n (%) | | | | | London & South  n(%) | | Midlands  n(%) | | | North  n(%) | | | |  |  |  |
|  |  | 1 | | | 58.1 (12.3) | | | | | 10.3 (11.5) | | | 3 (2.88) | | | | 4 (3.96) | | | | 2 (3.77) | | | | 5 (3.29) | | | | 7 (3.87) | | | | 0 | | | | | 5 (3.91) | | 1 (6.25) | | | 1 (1.64) | | | |  |  |  |
|  |  | 2 | | | 59.0 (13.4) | | | | | 11.1 (11.3) | | | 5 (4.81) | | | | 7 (6.93) | | | | 3 (5.66) | | | | 9 (5.92) | | | | 11 (6.08) | | | | 1 (4.17) | | | | | 9 (7.03) | | 1 (6.25) | | | 2 (3.28) | | | |  |  |  |
|  |  | 3 | | | 62.6 (9.8) | | | | | 6.0 (5.8) | | | 8 (7.69) | | | | 4 (3.96) | | | | 3 (5.66) | | | | 9 (5.92) | | | | 11 (6.08) | | | | 1 (4.17) | | | | | 3 (2.34) | | 1 (6.25) | | | 8 (13.11) | | | |  |  |  |
|  |  | 4 | | | 59.0 (13.8) | | | | | 9.6 (8.7) | | | 13 (12.50) | | | | 10 (9.90) | | | | 9 (16.98) | | | | 14 (9.21) | | | | 20 (11.05) | | | | 3 (12.50) | | | | | 11 (8.59) | | 2 (12.50) | | | 10 (16.39) | | | |  |  |  |
|  |  | 5 | | | 59.8 (10.9) | | | | | 7.6 (6.8) | | | 12 (11.54) | | | | 11 (10.89) | | | | 9 (16.98) | | | | 14 (9.21) | | | | 20 (11.05) | | | | 3 (12.50) | | | | | 8 (6.25) | | 4 (25.00) | | | 11 (18.03) | | | |  |  |  |
|  |  | 6 | | | 61.1 (12.0) | | | | | 5.5 (6.2) | | | 11 (10.58) | | | | 5 (4.95) | | | | 9 (16.98) | | | | 7 (4.61) | | | | 15 (8.29) | | | | 1 (4.17) | | | | | 2 (1.56) | | 3 (18.75) | | | 11 (18.03) | | | |  |  |  |
|  |  | 7 | | | 60.9 (10.8) | | | | | 7.0 (7.5) | | | 18 (17.31) | | | | 16 (15.84) | | | | 12 (22.64) | | | | 22 (14.47) | | | | 30 (16.57) | | | | 4 (16.67) | | | | | 17 (13.28) | | 4 (25.00) | | | 13 (21.31) | | | |  |  |  |
|  |  | 8 | | | 60.5 (10.1) | | | | | 6.5 (7.1) | | | 21 (20.19) | | | | 19 (18.81) | | | | 16 (30.19) | | | | 24 (15.79) | | | | 36 (19.89) | | | | 4 (16.67) | | | | | 20 (15.63) | | 5 (31.25) | | | 15 (24.59) | | | |  |  |  |
|  |  | 9 | | | 60.9 (10.0) | | | | | 7.5 (8.5) | | | 26 (25.00) | | | | 21 (20.79) | | | | 17 (32.08) | | | | 30 (19.74) | | | | 43 (23.76) | | | | 4 (16.67) | | | | | 22 (17.19) | | 7 (43.75) | | | 18 (29.51) | | | |  |  |  |
|  |  | 10 | | | 59.3 (11.2) | | | | | 6.8 (7.6) | | | 30 (28.85) | | | | 26 (25.74) | | | | 20 (37.74) | | | | 36 (23.68) | | | | 50 (27.62) | | | | 6 (25.00) | | | | | 30 (23.44) | | 8 (50.00) | | | 18 (29.51) | | | |  |  |  |
|  |  | 11 | | | 61.2 (10.0) | | | | | 7.7 (8.8) | | | 32 (30.77) | | | | 26 (25.74) | | | | 21 (39.62) | | | | 37(24.34) | | | | 54 (29.83) | | | | 4 (16.67) | | | | | 30 (23.44) | | 9 (56.25) | | | 19 (31.15) | | | |  |  |  |
|  |  | 12 | | | 62.9 (10.5) | | | | | 7.5 (8.2) | | | 12 (11.54) | | | | 7 (6.93) | | | | 10 (18.87) | | | | 9 (5.92) | | | | 19 (10.50) | | | | 0 | | | | | 6 (4.69) | | 6 (37.50) | | | 7 (11.48) | | | |  |  |  |
| **Non-Missing** | | 1 | | | 57.3 (12.0) | | | | | 8.1 (8.8) | | | 101 (97.12) | | | | 97 (96.04) | | | | 51 (96.23) | | | | 147 (96.71) | | | | 174 (96.13) | | | | 24 (100) | | | | | 123 (96.09) | | 15 (93.75) | | | 60 (98.36) | | | |  |  |  |
|  |  | 2 | | | 57.2 (11.9) | | | | | 8.0 (8.7) | | | 99 (95.19) | | | | 94 (93.07) | | | | 50 (94.34) | | | | 143 (94.08) | | | | 170 (93.92) | | | | 23 (95.83) | | | | | 119 (92.97) | | 15 (93.75) | | | 59 (96.72) | | | |  |  |  |
|  |  | 3 | | | 57.0 (12.0) | | | | | 8.3 (9.0) | | | 96 (92.31) | | | | 97 (96.04) | | | | 50 (94.34) | | | | 143 (94.08) | | | | 170 (93.92) | | | | 23 (95.83) | | | | | 125 (97.66) | | 15 (93.75) | | | 53 (86.89) | | | |  |  |  |
|  |  | 4 | | | 57.1 (11.8) | | | | | 8.0 (8.9) | | | 91 (87.50) | | | | 91 (90.10) | | | | 44 (83.02) | | | | 138 (90.79) | | | | 161 (88.95) | | | | 21 (87.50) | | | | | 117 (91.41) | | 14 (87.50) | | | 51 (83.61) | | | |  |  |  |
|  |  | 5 | | | 57.0 (12.1) | | | | | 8.3 (9.1) | | | 92 (88.46) | | | | 90 (89.11) | | | | 44 (83.02) | | | | 138 (90.79) | | | | 161 (88.95) | | | | 21 (87.50) | | | | | 120 (93.75) | | 12 (75.00) | | | 50 (81.97) | | | |  |  |  |
|  |  | 6 | | | 57.0 (11.9) | | | | | 8.4 (9.0) | | | 93 (89.42) | | | | 96 (95.05) | | | | 44 (83.02) | | | | 145 (95.39) | | | | 166 (91.71) | | | | 23 (95.83) | | | | | 126 (98.44) | | 13 (81.25) | | | 50 (81.97) | | | |  |  |  |
|  |  | 7 | | | 56.6 (12.1) | | | | | 8.4 (9.1) | | | 86 (82.69) | | | | 85 (84.16) | | | | 41 (77.36) | | | | 130 (85.53) | | | | 151 (83.43) | | | | 20 (83.33) | | | | | 111 (86.72) | | 12 (75.00) | | | 48 (78.69) | | | |  |  |  |
|  |  | 8 | | | 56.6 (12.3) | | | | | 8.6 (9.2) | | | 83 (79.81) | | | | 82 (81.19) | | | | 37 (69.81) | | | | 128 (84.21) | | | | 145 (80.11) | | | | 20 (83.33) | | | | | 108 (84.38) | | 11 (68.75) | | | 46 (75.41) | | | |  |  |  |
|  |  | 9 | | | 56.3 (12.3) | | | | | 8.4 (9.0) | | | 78 (75.00) | | | | 80 (79.21) | | | | 36 (67.92) | | | | 122 (80.26) | | | | 138 (76.24) | | | | 20 (83.33) | | | | | 106 (82.81) | | 9 (56.25) | | | 43 (70.49) | | | |  |  |  |
|  |  | 10 | | | 56.6 (12.2) | | | | | 8.7 (9.2) | | | 74 (71.15) | | | | 75 (74.26) | | | | 33 (62.26) | | | | 116 (76.32) | | | | 131 (72.38) | | | | 18 (75.00) | | | | | 98 (76.56) | | 8 (50.00) | | | 43 (70.49) | | | |  |  |  |
|  |  | 11 | | | 55.8 (12.4) | | | | | 8.4 (8.9) | | | 72 (69.23) | | | | 75 (74.26) | | | | 32 (60.38) | | | | 115 (75.66) | | | | 127 (70.17) | | | | 20 (83.33) | | | | | 98 (76.56) | | 7 (43.75) | | | 42 (68.85) | | | |  |  |  |
|  |  | 12 | | | 56.8 (12.0) | | | | | 8.3 (8.9) | | | 92 (88.46) | | | | 94 (93.07) | | | | 43 (81.13) | | | | 143 (94.08) | | | | 162 (89.50) | | | | 24 (100) | | | | | 122 (95.31) | | 10 (62.50) | | | 54 (88.52) | | | |  |  |  |
|  | |  | | |  | | | | | | | | | | | | | | | | | | | | | | | | | | | | | | | | | | | | | | | | |  |  |  |  |
|  | |  | | | **VAS** | | | | | | | | | | | | | | | | | | | | | | | | | | | | | | | | | | | | | | | | |  |  |  |  |
| **Missing** | | N^o^ Visits | | | Age (years) | | | Disease duration  (years) | | | | Treatment | | | | | | | | Gender | | | | | | | | Ethnicity | | | | | | | | NHS-Region | | | | | | | | | |  |  |  |  |
|  |  |  | | | Mean (SD) | | | Mean (SD) | | | | cDMARds  n(%) | | | | TNF  n(%) | | | | Male  n (%) | | | | Female  n(%) | | | | White  n(%) | | | | Other  n(%) | | | | London & South  n(%) | | | | Midlands  n(%) | | | | North  n(%) | |  |  |  |  |
|  |  | 1 | | | 58.9 (12.5) | | | 6.7 (8.8) | | | | 3 (2.88) | | | | 4 (3.96) | | | | 2 (3.77) | | | | 5 (3.29) | | | | 6 (3.31) | | | | 1 (4.17) | | | | 5 (3.91) | | | | 1 (6.25) | | | | 1 (1.64) | |  |  |  |  |
|  |  | 2 | | | 58.3 (12.4) | | | 8.7 (9.8) | | | | 5 (4.81) | | | | 5 (4.95) | | | | 3 (5.66) | | | | 7 (4.61) | | | | 9 (4.97) | | | | 1 (4.17) | | | | 8 (6.25) | | | | 1 (6.25) | | | | 1 (1.64) | |  |  |  |  |
|  |  | 3 | | | 62.6 (9.8) | | | 6.0 (5.8) | | | | 8 (7.69) | | | | 4 (3.96) | | | | 3 (5.66) | | | | 9 (5.92) | | | | 11 (6.08) | | | | 1 (4.17) | | | | 3 (2.34) | | | | 1 (6.25) | | | | 8 (13.11) | |  |  |  |  |
|  |  | 4 | | | 60.1 (13.1) | | | 10.3 (8.8) | | | | 12 (11.54) | | | | 9 (8.91) | | | | 8 (15.09) | | | | 13 (8.55) | | | | 19 (10.50) | | | | 2 (8.33) | | | | 10 (7.81) | | | | 2 (12.50) | | | | 9 (14.75) | |  |  |  |  |
|  |  | 5 | | | 59.8 (10.9) | | | 7.6 (6.8) | | | | 12 (11.54) | | | | 11 (10.89) | | | | 9 (16.98) | | | | 14 (9.21) | | | | 20 (11.05) | | | | 3 (12.50) | | | | 8 (6.25) | | | | 4 (25.00) | | | | 11 (18.03) | |  |  |  |  |
|  |  | 6 | | | 60.3 (12.0) | | | 5.8 (6.3) | | | | 10 (9.62) | | | | 5 (4.95) | | | | 8 (15.09) | | | | 7 (4.61) | | | | 14 (7.73) | | | | 1 (4.17) | | | | 1 (0.78) | | | | 3 (18.75) | | | | 11 (18.03) | |  |  |  |  |
|  |  | 7 | | | 60.8 (11.0) | | | 6.2 (6.1) | | | | 18 (17.31) | | | | 15 (14.85) | | | | 12 (22.64) | | | | 21 (13.82) | | | | 29 (16.02) | | | | 4 (16.67) | | | | 16 (12.50) | | | | 4 (25.00) | | | | 13 (21.31) | |  |  |  |  |
|  |  | 8 | | | 60.7 (10.2) | | | 5.8 (5.8) | | | | 21 (20.19) | | | | 17 (16.83) | | | | 16 (30.19) | | | | 22 (14.47) | | | | 34 (18.78) | | | | 4 (16.67) | | | | 18 (14.06) | | | | 5 (31.25) | | | | 15 (24.59) | |  |  |  |  |
|  |  | 9 | | | 60.8 (9.9) | | | 7.7 (8.5) | | | | 26 (25.00) | | | | 22 (21.78) | | | | 17 (32.08) | | | | 31 (20.39) | | | | 44 (24.31) | | | | 4 (16.67) | | | | 22 (17.19) | | | | 8 (50.00) | | | | 18 (29.51) | |  |  |  |  |
|  |  | 10 | | | 60.0 (10.8) | | | 6.8 (7.7) | | | | 29 (27.88) | | | | 25 (24.75) | | | | 19 (35.85) | | | | 35 (23.03) | | | | 48 (26.52) | | | | 6 (25.00) | | | | 29 (22.66) | | | | 8 (50.00) | | | | 17 (27.87) | |  |  |  |  |
|  |  | 11 | | | 61.3 (10.0) | | | 7.5 (8.8) | | | | 32 (30.77) | | | | 25 (24.75) | | | | 21 (39.62) | | | | 36 (23.68) | | | | 53 (29.28) | | | | 4 (16.67) | | | | 30 (23.44) | | | | 8 (50.00) | | | | 19 (31.15) | |  |  |  |  |
|  |  | 12 | | | 62.3 (10.8) | | | 8.2 (8.8) | | | | 9 (8.65) | | | | 7 (6.93) | | | | 8 (15.09) | | | | 8 (5.26) | | | | 16 (8.84) | | | | 0 | | | | 5 (3.91) | | | | 4 (25.00) | | | | 7 (11.48) | |  |  |  |  |
| **Non-Missing** | | 1 | | | 57.3 (12.0) | | | 8.2 (8.9) | | | | 101 (97.12) | | | | 97 (96.04) | | | | 51 (96.23) | | | | 147 (96.71) | | | | 175 (96.69) | | | | 23 (95.83) | | | | 123 (96.09) | | | | 15 (93.75) | | | | 60 (98.36) | |  |  |  |  |
|  |  | 2 | | | 57.3 (12.0) | | | 8.2 (8.8) | | | | 99 (95.19) | | | | 96 (95.05) | | | | 50 (94.34) | | | | 145 (95.39) | | | | 172 (95.03) | | | | 23 (95.83) | | | | 120 (93.75) | | | | 15 (93.75) | | | | 60 (98.36) | |  |  |  |  |
|  |  | 3 | | | 57.0 (12.0) | | | 8.3 (9.0) | | | | 96 (92.31) | | | | 97 (96.04) | | | | 50 (94.34) | | | | 143 (94.08) | | | | 170 (93.92) | | | | 23 (95.83) | | | | 125 (97.66) | | | | 15 (93.75) | | | | 53 (86.89) | |  |  |  |  |
|  |  | 4 | | | 57.0 (11.8) | | | 8.0 (8.8) | | | | 92 (88.46) | | | | 92 (91.09) | | | | 45 (84.91) | | | | 139 (91.45) | | | | 162 (89.50) | | | | 22 (91.67) | | | | 118 (92.19) | | | | 14 (87.50) | | | | 52 (85.25) | |  |  |  |  |
|  |  | 5 | | | 57.0 (12.1) | | | 8.3 (9.1) | | | | 92 (88.46) | | | | 90 (89.11) | | | | 44 (83.02) | | | | 138 (90.79) | | | | 161 (88.95) | | | | 21 (87.50) | | | | 120 (93.75) | | | | 12 (75.00) | | | | 50 (81.97) | |  |  |  |  |
|  |  | 6 | | | 57.1 (12.0) | | | 8.4 (9.0) | | | | 94 (90.38) | | | | 96 (95.05) | | | | 45 (84.91) | | | | 145 (95.39) | | | | 167 (92.27) | | | | 23 (95.83) | | | | 127 (99.22) | | | | 13 (81.25) | | | | 50 (81.97) | |  |  |  |  |
|  |  | 7 | | | 56.7 (12.1) | | | 8.6 (9.2) | | | | 86 (82.69) | | | | 86 (85.15) | | | | 41 (77.36) | | | | 131 (86.18) | | | | 152 (83.98) | | | | 20 (83.33) | | | | 112 (87.50) | | | | 12 (75.00) | | | | 48 (78.69) | |  |  |  |  |
|  |  | 8 | | | 56.6 (12.2) | | | 8.7 (9.3) | | | | 83 (79.81) | | | | 84 (83.17) | | | | 37 (69.81) | | | | 130 (85.53) | | | | 147 (81.22) | | | | 20 (83.33) | | | | 110 (85.94) | | | | 11 (68.75) | | | | 46 (75.41) | |  |  |  |  |
|  |  | 9 | | | 56.3 (12.4) | | | 8.3 (9.0) | | | | 78 (75.00) | | | | 79 (78.22) | | | | 36 (67.92) | | | | 121 (79.61) | | | | 137 (75.69) | | | | 20 (83.33) | | | | 106 (82.81) | | | | 8 (50.00) | | | | 43 (70.49) | |  |  |  |  |
|  |  | 10 | | | 56.4 (12.3) | | | 8.7 (9.2) | | | | 75 (72.12) | | | | 76 (75.25) | | | | 34 (64.15) | | | | 117 (76.97) | | | | 133 (73.48) | | | | 18 (75.00) | | | | 99 (77.34) | | | | 8 (50.00) | | | | 44 (72.13) | |  |  |  |  |
|  |  | 11 | | | 55.8 (12.3) | | | 8.5 (8.9) | | | | 72 (69.23) | | | | 76 (75.25) | | | | 32 (60.38) | | | | 116 (76.32) | | | | 128 (70.72) | | | | 20 (83.33) | | | | 98 (76.56) | | | | 8 (50.00) | | | | 42 (68.85) | |  |  |  |  |
|  |  | 12 | | | 56.9 (12.0) | | | 8.2 (8.9) | | | | 95 (91.35) | | | | 94 (93.07) | | | | 45 (84.91) | | | | 144 (94.74) | | | | 165 (91.16) | | | | 24 (100) | | | | 123 (96.09) | | | | 12 (75.00) | | | | 54 (88.52) | |  |  |  |  |
|  | | |  | | |  | | | | | | | | | | | | | | | | | | | | | | | | | | | | | | | | | | | | | | | | | |  |  |
|  | | |  | | | **DAS28** | | | | | | | | | | | | | | | | | | | | | | | | | | | | | | | | | | | | | | | | | |  |  |
| **Missing** | | | N^o^ Visits | | | Age (years) | | | Disease duration  (years) | | | | | | Treatment | | | | | | | | Gender | | | | | | | | Ethnicity | | | | | | | | NHS-Region | | | | | | | | |  |  |
|  |  |  |  | | | Mean (SD) | | | Mean (SD) | | | | | | cDMARds  n(%) | | | | TNF  n(%) | | | | Male  n(%) | | | | Female  n(%) | | | | White  n(%) | | | | Other  n(%) | | | | London & South  n(%) | | Midlands  n(%) | | | North  n(%) | | | |  |  |
|  |  |  | 1 | | | 58.9 (11.6) | | | 9.4 (10.8) | | | | | | 4 (3.85) | | | | 4 (3.96) | | | | 2 (3.77) | | | | 6 (3.95) | | | | 7 (3.87) | | | | 1 (4.17) | | | | 6 (4.69) | | 1 (6.25) | | | 1 (1.64) | | | |  |  |
|  |  |  | 2 | | | 59.0 (13.4) | | | 11.1 (11.3) | | | | | | 5 (4.81) | | | | 7 (6.93) | | | | 3 (5.66) | | | | 9 (5.92) | | | | 11 (6.08) | | | | 1 (4.17) | | | | 9 (7.03) | | 1 (6.25) | | | 2 (3.28) | | | |  |  |
|  |  |  | 3 | | | 63.0 (9.5) | | | 7.6 (7.7) | | | | | | 9 (8.65) | | | | 4 (3.96) | | | | 4 (7.55) | | | | 9 (5.92) | | | | 12 (6.63) | | | | 1 (4.17) | | | | 4 (3.13) | | 1 (6.25) | | | 8 (13.11) | | | |  |  |
|  |  |  | 4 | | | 58.0 (14.3) | | | 10.0 (8.6) | | | | | | 13 (12.50) | | | | 10 (9.90) | | | | 10 (18.87) | | | | 13 (8.55) | | | | 20 (11.05) | | | | 3 (12.50) | | | | 12 (9.38) | | 2 (12.50) | | | 9 (14.75) | | | |  |  |
|  |  |  | 5 | | | 59.8 (10.9) | | | 7.6 (6.8) | | | | | | 12 (11.54) | | | | 11 (10.89) | | | | 9 (16.98) | | | | 14 (9.21) | | | | 20 (11.05) | | | | 3 (12.50) | | | | 8 (6.25) | | 4 (25.00) | | | 11 (18.03) | | | |  |  |
|  |  |  | 6 | | | 61.1 (12.0) | | | 5.5 (6.2) | | | | | | 11 (10.58) | | | | 5 (4.95) | | | | 9 (16.98) | | | | 7 (4.61) | | | | 15 (8.29) | | | | 1 (4.17) | | | | 2 (1.56) | | 3 (18.75) | | | 11 (18.03) | | | |  |  |
|  |  |  | 7 | | | 60.9 (10.8) | | | 7.0 (7.5) | | | | | | 18 (17.31) | | | | 16 (15.84) | | | | 12 (22.64) | | | | 22 (14.47) | | | | 30 (16.57) | | | | 4 (16.67) | | | | 17 (13.28) | | 4 (25.00) | | | 13 (21.31) | | | |  |  |
|  |  |  | 8 | | | 60.5 (10.1) | | | 6.5 (7.1) | | | | | | 21 (20.19) | | | | 19 (18.81) | | | | 16 (30.19) | | | | 24 (15.79) | | | | 36 (19.89) | | | | 4 (16.67) | | | | 20 (15.63) | | 5 (31.25) | | | 15 (24.59) | | | |  |  |
|  |  |  | 9 | | | 60.8 (9.9) | | | 7.7 (8.5) | | | | | | 26 (25.00) | | | | 22 (21.78) | | | | 17 (32.08) | | | | 31 (20.39) | | | | 44 (24.31) | | | | 4 (16.67) | | | | 22 (17.19) | | 8 (50.00) | | | 18 (29.51) | | | |  |  |
|  |  |  | 10 | | | 59.3 (11.2) | | | 6.8 (7.6) | | | | | | 30 (28.85) | | | | 26 (25.74) | | | | 20 (37.74) | | | | 36 (23.68) | | | | 50 (27.62) | | | | 6 (25.00) | | | | 30 (23.44) | | 8 (50.00) | | | 18 (29.51) | | | |  |  |
|  |  |  | 11 | | | 61.2 (10.0) | | | 7.7 (8.8) | | | | | | 32 (30.77) | | | | 26 (25.74) | | | | 21 (39.62) | | | | 37 (24.34) | | | | 54 (29.83) | | | | 4 (16.67) | | | | 30 (23.44) | | 9 (56.25) | | | 19 (31.15) | | | |  |  |
|  |  |  | 12 | | | 62.9 (10.5) | | | 7.5 (8.2) | | | | | | 12 (11.54) | | | | 7 (6.93) | | | | 10 (18.87) | | | | 9 (5.92) | | | | 19 (10.50) | | | | 0 | | | | 6 (4.69) | | 6 (37.50) | | | 7 (11.48) | | | |  |  |
| **Non-Missing** | | | 1 | | | 57.3 (12.0) | | | 8.2 (8.8) | | | | | | 100 (96.15) | | | | 97 (96.04) | | | | 51 (96.23) | | | | 146 (96.05) | | | | 174 (96.13) | | | | 23 (95.83) | | | | 122 (95.31) | | 15 (93.75) | | | 60 (98.36) | | | |  |  |
|  |  |  | 2 | | | 57.2 (11.9) | | | 8.0 (8.7) | | | | | | 99 (95.19) | | | | 94 (93.07) | | | | 50 (94.34) | | | | 143 (94.08) | | | | 170 (93.92) | | | | 23 (95.83) | | | | 119 (92.97) | | 15 (93.75) | | | 59 (96.72) | | | |  |  |
|  |  |  | 3 | | | 57.0 (12.0) | | | 8.2 (8.9) | | | | | | 95 (91.35) | | | | 97 (96.04) | | | | 49 (92.45) | | | | 143 (94.08) | | | | 169 (93.37) | | | | 23 (95.83) | | | | 124 (96.88) | | 15 (93.75) | | | 53 (86.89) | | | |  |  |
|  |  |  | 4 | | | 57.3 (11.7) | | | 8.0 (8.9) | | | | | | 91 (87.50) | | | | 91 (90.10) | | | | 43 (81.13) | | | | 139 (91.45) | | | | 161 (88.95) | | | | 21 (87.50) | | | | 116 (90.63) | | 14 (87.50) | | | 52 (85.25) | | | |  |  |
|  |  |  | 5 | | | 57.0 (12.1) | | | 8.3 (9.1) | | | | | | 92 (88.46) | | | | 90 (89.11) | | | | 44 (83.02) | | | | 138 (90.79) | | | | 161 (88.95) | | | | 21 (87.50) | | | | 120 (93.75) | | 12 (75.00) | | | 50 (81.97) | | | |  |  |
|  |  |  | 6 | | | 57.0 (11.9) | | | 8.4 (9.0) | | | | | | 93 (89.42) | | | | 96 (95.05) | | | | 44 (83.02) | | | | 145 (95.39) | | | | 166 (91.71) | | | | 23 (95.83) | | | | 126 (98.44) | | 13 (81.25) | | | 50 (81.97) | | | |  |  |
|  |  |  | 7 | | | 56.6 (12.1) | | | 8.4 (9.1) | | | | | | 86 (82.69) | | | | 85 (84.16) | | | | 41 (77.36) | | | | 130 (85.53) | | | | 151 (83.43) | | | | 20 (83.33) | | | | 111 (86.72) | | 12 (75.00) | | | 48 (78.69) | | | |  |  |
|  |  |  | 8 | | | 56.6 (12.3) | | | 8.6 (9.2) | | | | | | 83 (79.81) | | | | 82 (81.19) | | | | 37 (69.81) | | | | 128 (84.21) | | | | 145 (80.11) | | | | 20 (83.33) | | | | 108 (84.38) | | 11 (68.75) | | | 46 (75.41) | | | |  |  |
|  |  |  | 9 | | | 56.3 (12.4) | | | 8.3 (9.0) | | | | | | 78 (75.00) | | | | 79 (78.22) | | | | 36 (67.92) | | | | 121 (79.61) | | | | 137 (75.69) | | | | 20 (83.33) | | | | 106 (82.81) | | 8 (50.00) | | | 43 (70.49) | | | |  |  |
|  |  |  | 10 | | | 56.6 (12.2) | | | 8.7 (9.2) | | | | | | 74 (72.15) | | | | 75 (74.26) | | | | 33 (62.26) | | | | 116 (76.32) | | | | 131 (72.38) | | | | 18 (75.00) | | | | 98 (76.56) | | 8 (50.00) | | | 43 (70.49) | | | |  |  |
|  |  |  | 11 | | | 55.8 (12.4) | | | 8.4 (8.9) | | | | | | 72 (69.23) | | | | 75 (74.26) | | | | 32 (60.38) | | | | 115 (75.66) | | | | 127 (70.17) | | | | 20 (83.33) | | | | 98 (76.56) | | 7 (43.75) | | | 42 (68.85) | | | |  |  |
|  |  |  | 12 | | | 56.8 (12.0) | | | 8.3 (8.9) | | | | | | 92 (88.46) | | | | 94 (93.07) | | | | 43 (81.13) | | | | 143 (94.08) | | | | 162 (89.50) | | | | 24 (100) | | | | 122 (95.31) | | 10 (62.50) | | | 54 (88.52) | | | |  |  |

TJC=tender joint counts; SJC=swollen joint counts; ESR=erythrocyte sedimentation rate; VAS = visual analogue scale; DAS28 = disease activity score (28 joints); SD=standard deviations; cDMARDs= combination disease modifying anti rheumatic drugs; TNFis = Tumor necrosis factor inhibitors

**Supplementary** **Figure 1: Percentage of dropouts in the components of the composite stratified by trial arm**


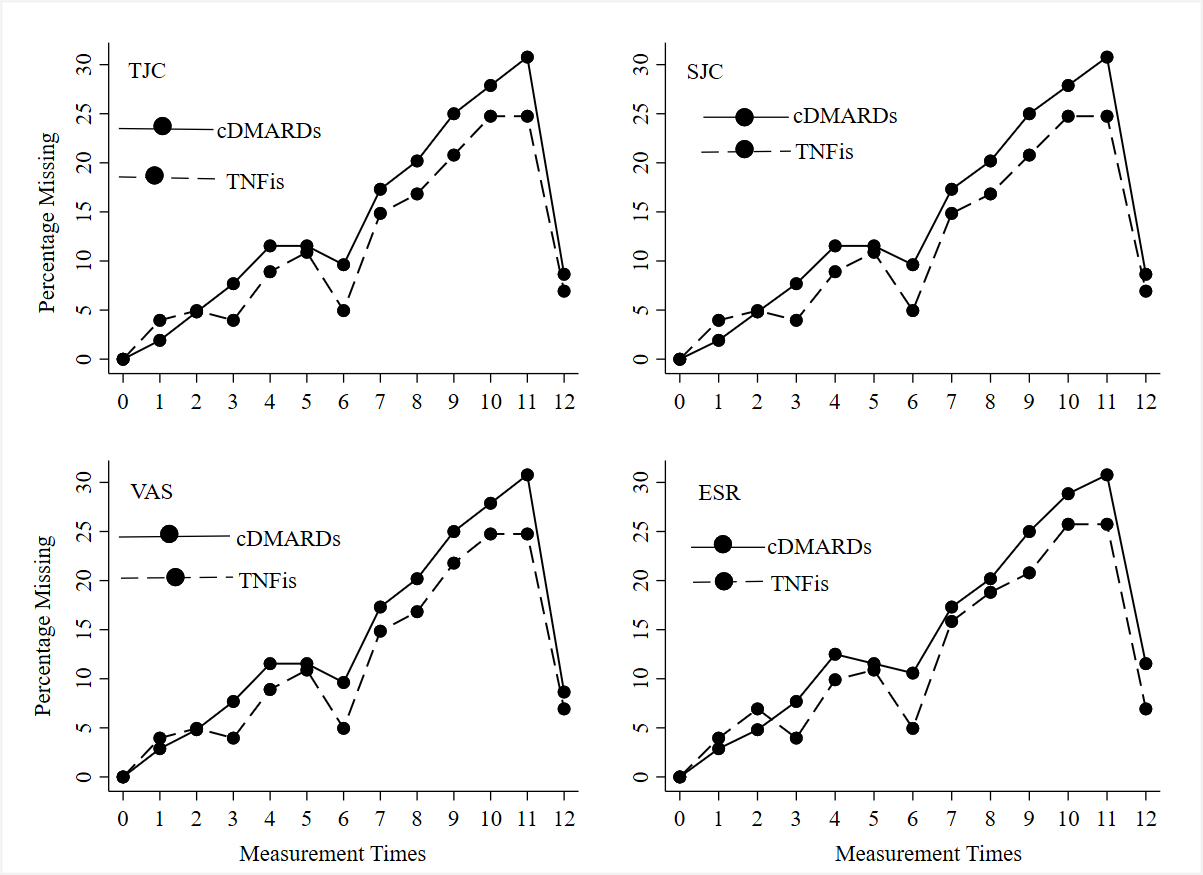


TJC=tender joint counts; SJC=swollen joint counts; ESR=erythrocyte sedimentation rate; VAS = visual analogue scale; cDMARDs= combination disease modifying anti rheumatic drugs; TNFis = Tumor necrosis factor inhibitors

References

1. Boers M and Tugwell P. The validity of pooled outcome measures (indices) in rheumatoid arthritis clinical trials. *The Journal of rheumatology* 1993; 20: 568-574.

2. Wolfe F, Michaud K, Pincus T, et al. The Disease Activity Score is not suitable as the sole criterion for initiation and evaluation of anti–tumor necrosis factor therapy in the clinic: discordance between assessment measures and limitations in questionnaire use for regulatory purposes. *Arthritis & Rheumatism* 2005; 52: 3873-3879.
